# Dynamic contrast-enhanced magnetic resonance imaging for pretreatment prediction of early chemo-radiotherapy response in larynx and hypopharynx carcinoma

**DOI:** 10.18632/oncotarget.12952

**Published:** 2016-10-27

**Authors:** Wei Guo, Dehong Luo, Xinyi Chen, Meng Lin, Lin Li, Yanfeng Zhao, Liang Yang, Lei Hu, Xinming Zhao, Chunwu Zhou

**Affiliations:** ^1^ Department of Diagnostic Radiology, Peking Union Medical College, Cancer Institute & Hospital, Chinese Academy of Medical Sciences, Beijing, China; ^2^ Department of Radiology, The Second Affiliated Hospital, Zhejiang University School of Medicine, Hangzhou, China

**Keywords:** chemo-radiotherapy, dynamic contrast-enhanced magnetic resonance imaging, larynx and hypopharynx carcinoma, semi-quantitative parameters

## Abstract

**Purpose:**

This study is to investigate the use of dynamic contrast-enhanced magnetic resonance imaging in predicting early response to CRT (chemo-radiotherapy) in patients with larynx and hypopharynx carcinoma from primary tumors.

**Method:**

Sixty-two patients with larynx and hypopharynx carcinoma underwent two DCE-MRI studies: a baseline exam before any treatmentanda post-treatment exam 3 weeks after CRT. At the end of treatment, patients were classified as responders, or non-responders according to the Response Evaluation Criteria in Solid Tumors criteria (RECIST). The time intensity curves (TIC) were extracted and processed to obtain time to peak (TTP), maximum slope of increase (MSI), maximum slope of decrease (MSD) and positive enhancement integral (PEI), and the semi-quantitative MRI parameters were compared and analyzed between the two groups.

**Results:**

Fifty-four and 8 patients were included the responder and non-responder groups. It was observed that the MSI, MSD, and PEI were significantly lower post-treatment than pre-treatment(*P* < 0.05). The pretreatment MSI, MSD, and PEI parameters of responders were significantly higher than those of non-responders (*P*< 0.05). The post-treatment MSI, MSD, and PEI parameters of responders were significantly lower than those of non-responders (*P*< 0.05). Based on ROC curve analysis, at a threshold of 154.81 for pretreatment MSI, the corresponding AUC, sensitivity, and specificity were 0.882, 89.3% and 73.5%, respectively.

**Conclusion:**

The semi-quantitative DCE-MRI may aid in the prediction of early response to CRT in patients with larynx and hypopharynx carcinoma.

## INTRODUCTION

Larynx and hypopharynx carcinoma are common malignant neoplasms in head and neck squamous cell carcinoma (HNSCC). The incidence of hypopharynx carcinoma was lower than that of larynx carcinoma. As the disease advances there is an increase in comorbidities. The overall survival is relatively poor because of high rates of regional recurrence and distant metastasis [1].

Radiation therapy and concurrent or induction chemotherapy is the standard treatment for organ preservation in larynx and hypopharynx carcinoma [2–3]. However, not all patients with larynx and hypopharynx carcinoma respond to chemo-radiotherapy. Resistance to chemo-radiotherapy is widely recognized as the main cause of relapse for larynx and hypopharynx carcinoma. Biomarkers that may predict treatment outcome and hence stratify patients that would benefit from chemo-radiation therapy is thus of great clinical significance. For non-responders, alternative treatment strategies such as upfront neck surgery and novel treatment modalities that include monoclonal antibodies, molecular inhibitors, and gene therapy can be individually tailored to improve survival and quality of life [4].

Dynamic contrast enhanced MRI (DCE-MRI) better reveals the relationship between blood perfusion and tumor hypoxia as compared to alternative imaging methods such as DWI, MRS, and PET-CT [5–6]. Angiogenetic alterations cause changes in the parameters of vascular physiology (perfusion, blood volume, and capillary permeability) and DCE MRI is reveals the progress of angiogenesis and provides information of vascularization at the tissue level. DCE-MRI holds promise for predicting and monitoring treatment response for HNSCC, several DCE-MRI studies have shown favorable outcomes for HNSCC with high vascularity on the pre-treatment DCE-MRI [8–14], and however few studies studied the DCE-MRI derived parameters on larynx and hypopharynx carcinoma.

The current study was aimed to investigate whether semi-quantitative analysis of DCE-MRI data from primary tumors may provide useful information regarding larynx and hypopharynx carcinoma response to chemo-radiotherapy.

## RESULTS

### Different therapy regimens pre-treatment

The pre-treatment TTP, MSI, MSD, and PEI for RT group were 91.50±21.40 seconds, 159.77±71.74, 75.86± 45.74, and 141.57±75.84, respectively; for concurrent CRT group were 83.00±19.35 seconds, 172.11±91.93, 110.78±27.93, and 155.65±62.85, respectively; for IC followed concurrent CRT group were 81.38±18.18 seconds, 181.32±80.98, 97.93±93.71, and 172.27±91.77, respectively (*P* = 0.403, 0.787, 0.713, and 0.566, respectively, Table 1).

**Table 1:** Pretreatment DCE-MRI semi-quantitative parameters for the different therapy regimens

DCE-MRI Parameters	RT (*n*= 8)	Concurrent CRT (*n*= 20)	IC+ConcurrentCRT (*n*= 34)	F	*P*
TTP(s)	91.50±21.40	83.00±19.35	81.38±18.18	0.923	0.403
MSI	159.77±71.74	172.11±91.93	181.32±80.98	0.240	0.787
MSD	75.86± 45.74	110.78±27.93	97.93±93.71	0.340	0.713
PEI	141.57±75.84	155.65±62.85	172.27±91.77	0.576	0.566

### Different response groups pre-treatment

**Figure 1:**
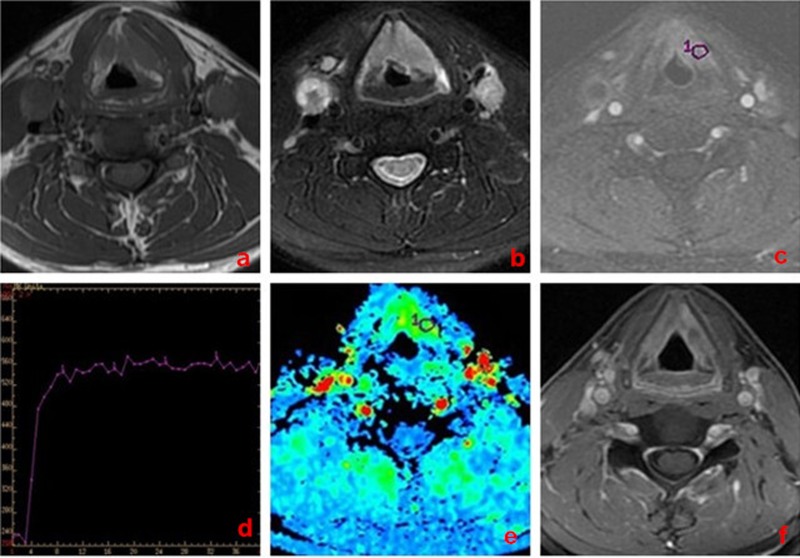
A 58-year-old man with larynx carcinoma, good response to chemo-radiotherapy **a**. Transverse T_1_-weighted imaging. **b**. Transverse T_2_-weighted imaging. **c**. DCE-MRI map. **d**. TIC map. **e**.The MSI map, MSI value was calculated as 168.74. **f**. Transverse T_1_-weighted enhancement imaging show that tumor mass was reduced obviously (> 50% reduction) after treatment.

**Figure 2:**
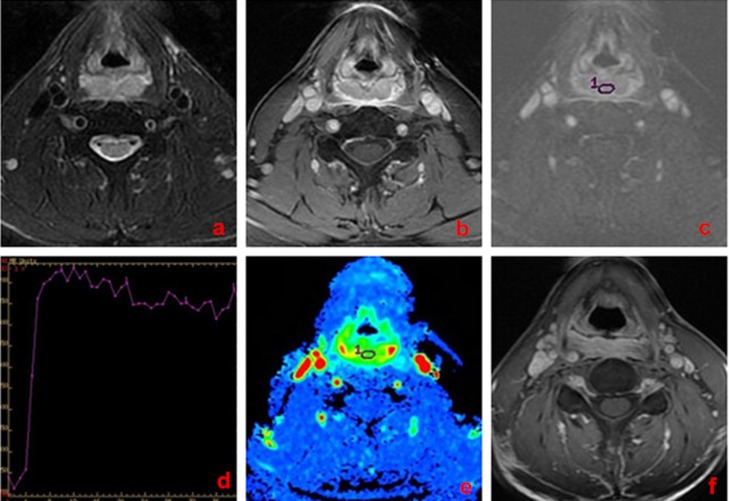
A 62-year-old man with hypopharynx carcinoma, poor response to chemo-radiotherapy **a**. Transverse T_2_-weighted imaging. **b**. Transverse T_1_-weighted enhancement imaging. **c**. DCE-MRI map. **d**. TIC map. **e**.The MSI map, MSI value was calculated as 65.72. **f**. Transverse T_1_-weighted enhancement imaging show that tumor mass was slightly reduced (< 30% reduction) after treatment.

54 of 62 patients (87.1%) were categorized into the responder group (CR, *n* = 28; PR, *n* = 26; Figure 1) whereas the other 8 were considered non-responders (SD, *n* = 8; Figure 2) at the end of treatment. For primary tumors, the pretreatment TTP, MSI, MSD, and PEI for responders were 80.96±17.59seconds, 227.49±70.58, 130.69±126.99, and 198.39±92.18, respectively; for non-responders they were 85.06 ± 22.05 seconds, 126.92±45.15, 73.32±64.33, and 133.76±57.05, respectively (*P* = 0.401, 0.000, 0.025, and 0.001, respectively, Table 2).

**Table 2:** Pretreatment DCE-MRI semi-quantitative parameters for the responders and non-responders

DCE-MRI Parameters	Responders (*n*= 54)	Non-responders (*n* = 8)	Z	*P*
TTP(s)	80.96±17.59	85.06±22.05	−0.845	0.401
MSI	227.49±70.58	126.92±45.15	6.796	0.000
MSD	130.69±126.99	73.32±64.33	2.303	0.025
PEI	198.39±92.18	133.76±57.05	3.380	0.001

### Different response groups post-treatment

For primary tumors, the post-treatment TTP, MSI, MSD, and PEI for responders were 75.06±13.83seconds, 32.31±43.78, 46.71±56.33, and 78.63±12.33, respectively; for non-responders they were 76.33 ± 21.23 seconds, 79.33±65.07, 62.24±57.57, and 102.11±27.11, respectively (*P* = 0.198, 0.000, 0.026, and 0.002, respectively, Table 3).

**Table 3:** Posttreatment DCE-MRI semi-quantitative parameters for the responders and non-responders

DCE-MRI Parameters	Responders (*n*= 54)	Non-responders (*n*= 8)	Z	*P*
TTP(s)	75.06±13.83	76.33±21.23	1.348	0.198
MSI	32.31±43.78	79.33±65.07	3.914	0.000
MSD	46.71±56.33	62.24±57.57	7.540	0.026
PEI	78.63±12.33	102.11±27.11	−5.517	0.002

### Pre- and post- treatment comparison

For primary tumors, the MSI, MSD and PEI parameters in post-treatment group was significantly lower than in pre-treatment group (*P* = 0.000, 0.003, 0.013; Table 4), whereas the TTP parameters of pre- and post- treatment showed no significant changes (*P* = 0.223; Table 4).

**Table 4:** Comparison of DCE-MRI semi-quantitative parameters between pre and post treatment

Group		Parameters		
	TTP(s)	MSI	MSD	PEI
Pretreatment (*n* = 62)	82.33±16.18	181.54±55.73	102.46±77.56	178.66±88.52
Posttreatment (*n* = 62)	77.64±17.22	65.44±48.97	57.56±48.77	93.35±22.03
*t*	−1.257	4.183	−3.477	−1.018
*P*	0.223	0.000	0.003	0.013

### Differentiation of responders from non-responders

**Figure 3:**
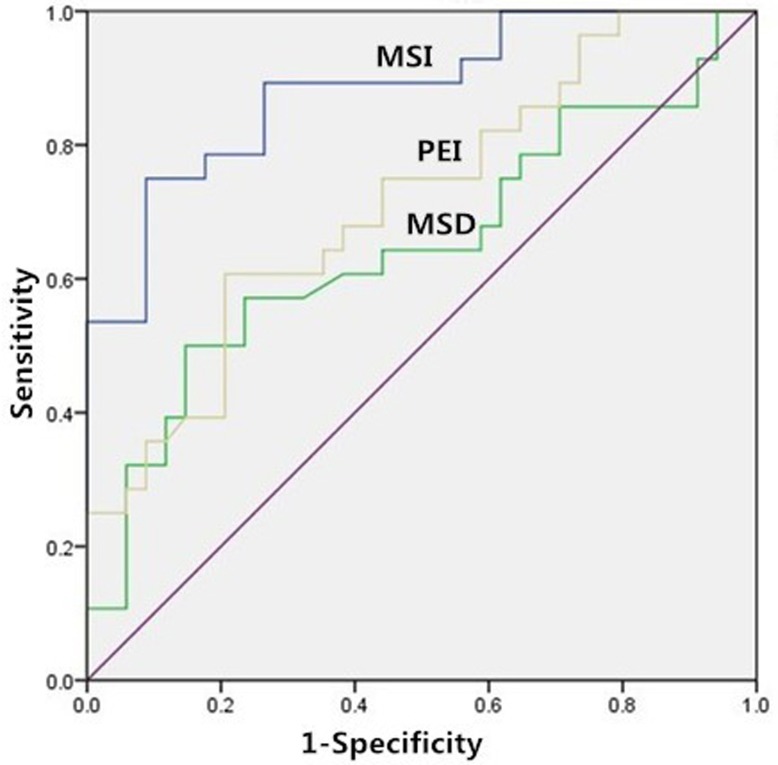
Receiver-operating characteristics curve Receiver operating characteristic curve for the pre-treatment MSI, MSD and PEI, for distinguishing between responders and non-responders.

The ROC curve analysis indicated that, the areas under curve (AUC) of pre-treatment MSI, MSD, PEI and post-treatment MSI, MSD, PEI were 0.882, 0.802, 0.758, 0.798, 0.721 and 0.678, respectively. The cutoff for pretreatment MSI value in best predicting responders was 154.81 and the corresponding sensitivity, and specificity were 89.3% and 73.5%, respectively (Figure 3).

## DISCUSSION

In this study, the use of semi-quantitative parameters derived from DCE-MR imaging data for prediction of treatment response to chemo-radiation therapy in patients with larynx and hypopharynx carcinoma was investigated. Our preliminary results indicated that higher pre-treatment MSI, MSD, PEI and lower post-treatment MSI, MSD, PEI may be associated with good response to chemo-radiotherapy in larynx and hypopharynx carcinoma. While pre- and post- treatment TTP both did not differ significantly between the responders and non-responders. The ROC curve analysis indicated that, when both sensitivity and specificity were optimized, the threshold of pretreatment MSI value in best predicting tumor’s chemotherapeutic response was 154.81 and the corresponding AUC, sensitivity, and specificity were 0.882, 89.3% and 73.5%, respectively. These results suggest that the semi-quantitative DCE-MRI parameters have a potential as prognostic biomarkers for predicting the early therapeutic response in larynx and hypopharynx carcinoma.

Past studies of pre-treatment DCE-MRI showed that hypoxia or low perfusion pre-therapy are markers of poor response and prognosis, however mostly focused on quantitative metrics [18–25]. The drawback of quantitative measurements is the excessive computation needed and more importantly fully quantitative metrics may be impacted by the dynamic nature of the data acquisition and need of accurate timing and pathological information. On the other hand, semi-quantitative DCE-MRI parameters including PEI, MSI and MSD are based on intuitive and simplistic model that reflect a combination of tumor blood volume and blood flow and are independent of the arterial blood flow. Previous studies have shown that semi-quantitative and quantitative parameters and are highly correlated [26], and even in some cases semi-quantitative parameters were superior to fully quantitative counterparts in predicting response and prognosis [27–29].

In the current study, patients with disease responsive to chemo-radiation therapy was observed to have significantly higher pre-treatment and significantly lower post-treatment MSI, MSD and PEI in primary tumor regions as compared to patients with no response. These results are consistent with the idea that vascularization at the tissue level and tumor hypoxia in solid tumor may be closely related to chemo-radiotherapy sensitivity [30–31]. Lower TTP values were also observed both pre- and post- treatment in primary tumors regions of responders as compared to those in non-responders, however without statistical significance (*P* > 0.05). The results in this study are in line with previous reports and support the hypothesis that tumors with relatively higher blood volume and blood flow are associated with increased oxygenation levels leading to better access to chemo-radiation [5]. On the other hand, tumor hypoxia adversely influences treatment response [19]. In a recent DCE-MRI study [32], an inverse correlation was observed between semi-quantitative parameters of DCE-MRI {maximal enhancement ratio (MER), slope of enhancement (SLE) } and hypoxia in in a maxillofacial VX2 rabbit model; this finding suggests that high tumor blood flow and volume are associated with low levels of hypoxia.

In addition, DCE-MRI parameters can also be confounded by tumors therapy regimens. Different therapy regimens may have play a confounding role in differentiating responders from non-responders. To account for these differences, we performed an analysis of subgroups of patients who underwent RT, Concurrent CRT and IC followed Concurrent CRT. There were no significant differences found for all pre-treatment DCE-MRI parameters the different therapy regimens. These above findings suggest therapy regimens did not have significant influence in distinguishing between responders and non-responders.

There are several limitations in this study. Firstly, this study only evaluated the short-term response rather than the long-term survival outcomes with disease-free survival status. Therefore, future studies with long-term follow-up survival outcome are desired for further confirmation. Secondly, a validation cohort is desirable for improved completeness of studies with predictive outcomes. This is however limited by the patient group available in this study. Thirdly, only the pre-treatment DCE-MRI parameters were analyzed, but the changes of these parameters pre- and post- treatment were not compared. This would be desirable for overall exploration of the utilities of the semi-quantitative DCE MRI parameters. Lastly, it would be interesting to have comparison between semi-quantitative and fully quantitative parameters in this case.

## CONCLUSIONS

Semi-quantitative DCE-MRI parameters, especially the pretreatment MSI measurements, show significant difference between responders and non-responders in larynx and hypopharynx carcinoma and may thus be used for predicting the treatment outcomes.

## MATERIALS AND METHODS

### Patients and treatment

This retrospective study was approved by the ethics committee of Cancer Institute & Hospital, Chinese Academy of Medical Sciences. All patients provided written informed consent.

Out of the 68 patients who met the inclusion criteria (from December 2013 to October 2015), 62 patients (57 male, 5female with a mean range of 45-72 years; mean age, 55.8±17.4 years) were recruited into this study, and the other 6 were excluded due to severe image distortions (*n* = 2) or the length-diameter of primary lesions < 1.0cm (*n* = 4). The primary tumor locations at initial presentation were pyriform sinus (*n* = 34), posterior pharyngeal wall (*n* = 8), the post-cricoid region (*n* = 4), glottic region (*n* = 6), supraglottic region (*n* = 8), and infraglottic region (*n* = 2). Distributions of clinical staging (TNM) were detailed as follow: T1, *n* = 3 (3/62, 4.84%); T2, *n* = 12 (12/62, 19.35%); T3, *n* = 27 (27/62, 43.55%); T4, *n* = 20 (20/62, 32.26%); N0, *n* = 6 (6/62, 9.68%); N1, *n* = 17 (17/62, 27.42%); N2, *n* = 27 (27/62, 43.55%); N3, *n* = 12 (12/62, 19.35%); M0, *n* = 59 (59/62, 95.16%); M1, *n* = 3 (3/62, 4.84%). The tumor length-diameter with a mean range of 1.5-7.8 cm; mean size, 4.2±3.6 cm. All recruited patients eligible for this study met the following criteria: confirmed diagnosis of squamous cell carcinoma (SCC) by biopsy; MR safe (no metal prosthesis, cardiac pacemaker, metal internal stent, etc.); not allergic to gadolinium-based contrast agent; have received chemo-radiotherapy.

The radiation therapy regimen (*n* = 8) included a total dosage of 7040 cGy that was given in 32 fractions at a daily dose of 220 cGy per fraction over a course of 42 days. The chemotherapy regimen was variable and included either concurrent chemotherapy alone (*n* = 20) or induction chemotherapy followed by concurrent chemotherapy (*n* = 34). Induction chemotherapy regimen included paclitaxel (270 mg/m^2^ of body-surface area, for day 1), followed by intravenous cisplatin (40 mg/m^2^, for day 1-2). Induction chemotherapy was given every 3 weeks for two cycles. Concurrent chemotherapy was administrated with cisplatin (30 mg/m^2^) weekly, and was given for a maximum of seven weekly doses during the course of radiotherapy.

All patients underwent 2 MRI exams, first exam was performed prior to any treatment at the baseline and second study was performed within 3 weeks after completion of CRT. At the end of the treatment, patients were evaluated and classified as responders or non-responders according to the Response Evaluation Criteria in Solid Tumors (RECIST) criteria, based on their MRI measurement^15^. Patients were considered as responders when all assessable tumors completely disappeared or partially reduced (≥ 30% of in the sum of maximal diameters) on the follow-up MRI. On the contrary, patients were considered non-responders if measurable tumors were relatively stable (< 30% reduction or < 20% increase in the sum of maximal diameters) or progressed (≥ 20% increase of original tumor or appearance of new lesions).

### Imaging acquisition

Head and neck MRI exam was conducted on a 3.0-T scanners (Discovery 750, GE, WI, USA, *n* = 36 or Signa Excite, GE, WI, USA, *n* = 26) equipped with an 8-channel neurovascular phased-array coil. Before any anti-tumor treatments, all the enrolled subjects received the following conventional MRI sequences: axial T_1_-weighted imaging with fast spin echo (Ax T_1_WI-FSE): repetition time/echo time (TR/TE) = 660/9.3ms, FOV = 260 × 260 mm^2^, reconstruction matrix = 960× 960, slice number = 30, slice thickness/gap = 4/0.4 mm; axial T2-weighted imaging with fast spin echo (Ax T_2_WI-FSE): TR/TE = 5760/88.3ms, FOV = 260 × 260 mm^2^, reconstruction matrix = 960× 960, slice number = 30, slice thickness/gap = 4/0.4 mm.

DCE-MRI was performed using LAVA-XV sequence (liver acquisition with volume acceleration-extended volume). The DCE-MRI parameters were: TR/TE of 2.8/1.3ms, FOV of 26 cm^2^, temporal resolution = 7 s/dynamic, slice thickness/gap = 4.2/0 mm, FA of 15°, and receiver bandwidth of 510 Hz/pixel. 40 phases were performed and total scan time was approximately 280 seconds.

Before the contrast media injection, baseline images were obtained. After initiation of DCE acquisition, Gadodiamide injection (Omniscan, GE, Ireland) was intravenously injected at a dose of 0.1mmol/kg of body weight with a rate of 2.0ml/s, followed by a 20 ml saline flush with a power injector. Patients were instructed not to swallow, move their tongues, open their mouths, or make any other voluntary motion during the DCE-MRI acquisition. Following the DCE-MRI scan, post-contrast enhanced anatomical T1-weighted images were acquired as a part of the routine clinical examination.

### Image processing

Time intensity curves (TIC) were also obtained, and time to peak (TTP), maximum slope of increase (MSI), maximum slope of decrease (MSD) and positive enhancement integral (PEI) were calculated as previously described [16–17]. DCE-derived parameters were calculated and measured blindly by one radiologists with 5-years of experience in head and neck imaging. The regions of interest (ROIs) were manually placed on each tumor area, avoiding visually large cystic or necrotic areas. The TTP, MSI, MSD and PEI values in each ROI were calculated and the average over all three ROIs were used for analysis, and ROI area was 30~50 mm^2^.

### Statistical analysis

All measurements are presented as mean ± standard deviation (mean ± SD). Normality test for DCE-MRI parameters were done using the Kolmogorov-Smirnov test. The Student *t* test and Mann-Whitney U tests were used to compare the pre- and post-treatment DCE parameters (TTP, MSI, MSD and PEI), and parametric differences were tested using the statistical method of analysis of variance (ANOVA). ROC analyses were also used to ascertain the best discriminatory model in differentiating responders from non-responders. Diagnostic accuracy was determined using the values of area under curve (AUC), and the predicting cutoff value, sensitivity, and specificity were calculated as well. All statistical analyses were performed using SPSS (version19; IBM SPSS; Chicago, IL), with a 2-tailed probability value, *P* < 0.05 was considered statistically significant.
